# Finding a Balance: A Systematic Review of the Biomechanical Effects of Vestibular Prostheses on Stability in Humans

**DOI:** 10.3390/jfmk5020023

**Published:** 2020-03-30

**Authors:** Felix Haxby, Mohammad Akrami, Reza Zamani

**Affiliations:** 1 Medical School, University of Exeter, Exeter EX1 2LU, UK; felixhaxby@gmail.com (F.H.); r.zamani@exeter.ac.uk (R.Z.); 2 Department of Engineering, College of Engineering, Mathematics, and Physical Sciences University of Exeter, Exeter EX4 4QF, UK

**Keywords:** balance, vestibular prostheses, biomechanics, stability, review

## Abstract

The vestibular system is located in the inner ear and is responsible for maintaining balance in humans. Bilateral vestibular dysfunction (BVD) is a disorder that adversely affects vestibular function. This results in symptoms such as postural imbalance and vertigo, increasing the incidence of falls and worsening quality of life. Current therapeutic options are often ineffective, with a focus on symptom management. Artificial stimulation of the vestibular system, via a vestibular prosthesis, is a technique being explored to restore vestibular function. This review systematically searched for literature that reported the effect of artificial vestibular stimulation on human behaviours related to balance, using the Preferred Reporting Items for Systematic Reviews and Meta-Analyses (PRISMA) technique. A total of 21 papers matched the inclusion criteria of the literature search conducted using the PubMed and Web of Science databases (February 2019). The populations for these studies included both healthy adults and patients with BVD. In every paper, artificial vestibular stimulation caused an improvement in certain behaviours related to balance, although the extent of the effect varied greatly. Various behaviours were measured such as the vestibulo-ocular reflex, postural sway and certain gait characteristics. Two classes of prosthesis were evaluated and both showed a significant improvement in at least one aspect of balance-related behaviour in every paper included. No adverse effects were reported for prostheses using noisy galvanic vestibular stimulation, however, prosthetic implantation sometimes caused hearing or vestibular loss. Significant heterogeneity in methodology, study population and disease aetiology were observed. The present study confirms the feasibility of vestibular implants in humans for restoring balance in controlled conditions, but more research needs to be conducted to determine their effects on balance in non-clinical settings.

## 1. Introduction

The vestibular system is the fundamental mediator of dynamic behaviour. It facilitates unconscious, impulsive control of compensatory movements such as postural and ocular reflexes [1]. The coordination of these reflexes results in what is often considered the sixth sense: balance [2]. The peripheral vestibular system, along with the cochlea, is located in the labyrinth of each inner ear. The semi-circular canals function as a gyroscope, communicating information on the movements of the head to specific effectors, while the otoliths organs can be compared to accelerometers. Both rotational movements and linear accelerations need to be detected to accurately coordinate movement with balance, and the relationship of the vestibular apparatus is reciprocal between the two ears [3]. 

Detection of movement is accomplished through two classes of end-organ: the semi-circular canals (SCCs) and the otolith organs [4]. The orthogonal arrangement of the three SCCs allows the detection of rotational movement in three dimensions [5]. The SCCs elicit the vestibular-ocular reflex (VOR), which is responsible for stabilising vision and contributing to balance control [5]. In contrast, linear acceleration is detected by the otolith organs, comprised of the utricle and saccule [6]. The otolith organs play a central role in the postural-righting reflexes of the neck, trunk and legs [6]. Synergistic processing and coordination of these vestibular signals, in combination with inputs from the visual and proprioceptive systems, results in the ability to balance [5]. 

### 1.1. Vestibular Dysfunction

Bilateral vestibular disorder (BVD) is an umbrella term used to describe individuals with bilaterally diminished peripheral vestibular function [2]. It is highly heterogeneous; age-related deterioration of the vestibular system is inevitable, and disorders such as benign paroxysmal positional vertigo, meningitis and Ménière’s disease can all be contributary factors [2,7]. Treatment with antibiotics (such as Gentamicin) and trauma-related injury can also cause BVD. However, idiopathic BV is the diagnosis in ~50% of cases [8]. Symptoms of BVD include oscillopsia, vertigo, headaches, nystagmus and chronic imbalance [2]. These are attributable to severely reduced or absent function of vestibular nerves, end organs or both [9]. Diminished function of the vestibular system adversely affects the VOR and postural-righting reflexes, which reduces ability to balance in both static and dynamic situations. 

BVD has an adverse effect on quality of life; 84% of patients reported a significant decrease in their quality of life and one study reported a 31-fold increase in the risk of falls [9]. Patients commonly quote a lack of independence, inability to drive and the need for assistance with daily activities as prevalent negative factors [10]. Socioeconomic factors are also affected due to a significant increase in healthcare utilisation and a loss of patient productivity [11]. Although the complete loss of vestibular function is very rare, improvements in vestibular function after diagnosis happen in <20% of cases [12].

Current treatment options are limited as there is no specific medical or behavioural therapy that have been shown to restore vestibular function in the long-term [8]. Vestibular rehabilitation therapy is often prescribed with the aim of symptom relief [12]. However, a positive response to this is normally limited to patients with unilateral vestibulopathy and this treatment is ineffective in improving response to unpredictable or high-frequency movements [12]. Additionally, the time required to participate in physical therapy, and the delay between therapy initiation and outcome improvement, highlights the need for more comprehensive treatment options [13]. Attempts to develop a viable prosthetic device to restore vestibular function have gained traction and have been evidenced to improve VOR, postural stability and gait speed in humans [14,15,16]. The successful development of a prosthetic device that restores vestibular function would confer great benefit on patients; improving their quality of life and reducing the frequency of falls and hospital visits [17]. The potential economic value in such a device is noteworthy; a recent study conducted a sensitivity analysis, predicting that a device recovering 75% of vestibular function would have a cost-utility of $37986/quality-adjusted life year (QALY), well below the $50,000/QALY willingness-to-pay threshold at which medical interventions are considered to be ‘highly cost-effective’ in the US [18]. Additionally, there are numerous diseases that list vertigo or chronic imbalance as a symptom, thus, developing a successful vestibular prosthesis has the potential to cause a wide-reaching therapeutic impact. 

### 1.2. Prosthetic Devices

Vestibular prostheses aim to artificially stimulate vestibular afferents in a parametrically controllable fashion [19]. This may result in the restoration of vestibular function, and consequently, balance control. In order to evaluate the effectiveness of prosthetic devices, changes in human behaviours related to balance can be measured before and after stimulation. The VOR, postural sway and gait speed are all behaviours that contribute to balance and that rely on sensory input from the vestibular system [5]. All three behaviours are also quantifiable and are therefore commonly used to assess the effectiveness of different prostheses [9]. Two different mechanisms are explored as a potential means of artificial vestibular stimulation which are vestibular implants and galvanic vestibular stimulation (GVS). 

#### 1.2.1. Vestibular Implants

Vestibular implant prototypes aim to artificially stimulate the vestibular nerves, closely replicating the natural input of a healthy vestibular system [20]. Typically, this device consists of an external component, fixed to the patient’s head, that senses radial and linear acceleration and transforms this into an electrical pattern of biphasic, charge-balanced currents via a processor [20]. This current is transmitted to the vestibular nerve via stimulation of electrodes placed in the vicinity of the ampullary nerve endings or within the SCCs in proximity to the ampullae themselves. Stimulation of SCC afferents has been shown to restore VOR by inducing canal-specific eye movements, with modulation of amplitude or frequency causing a proportional effect on the velocity and direction of the movement [21]. In order to restore bi-directional eye movements using unilateral stimulation, an artificial ‘spontaneous’ impulse firing rate (as close as possible to the 90 spikes per second firing rate produced in a healthy vestibular system) must be induced in each vestibular nerve [22]. This can then be either up- or downregulated, in response to specific movements to elicit eye movements in different directions [21]. Postural responses due to artificial stimulation of the vestibular nerve in humans have also been experimentally elucidated [15]. The technology used in designing vestibular implants is closely aligned with that of cochlear implants. In fact, the majority of vestibular implants tested on humans have been modified from cochlear implants [20].

A myriad of options are available when designing this type of implant. There have been multiple signal-processing strategies, sensor fixation techniques and both multi-channel and single-channel arrays developed [20]. Moreover, there is a choice in surgical implantation approach: access to the superior (SAN), lateral (LAN) and posterior (PAN) ampullary nerves can be achieved via extralabyrinthine stimulation, whereas access to the ampullae of the SCCs requires more invasive, intralabyrinthine stimulation [19]. Both these approaches have been used to successfully implant a vestibular prosthesis in humans [19,20]. Loss of hearing is a risk for both methods and although hearing preservation has been observed in studies on primates, in the vast majority of studies on humans, the recipients were bilaterally deaf meaning the likelihood of retaining hearing is yet to be clarified [23].

Due to the limited number and significant heterogeneity of relevant trials conducted on humans so far, it is difficult to draw conclusions about the relative strengths of these design choices. Therefore, the present review will focus on synthesising quantitative results of behaviours affecting balance in humans following artificial vestibular stimulation.

#### 1.2.2. Galvanic Vestibular Stimulation (nGVS)

Artificial control over vestibular afferents can also be achieved via galvanic vestibular stimulation [24]. This method is non-invasive and modulates afferent firing rates in the otolith organs and the SCC afferents transcutaneously [24] which showed that constant galvanic vestibular stimulation can adversely affect unilateral postural or oculomotor function. The use of zero-mean current noisy galvanic stimulation (nGVS) is not consequently considered more advantageous. Battery-powered portable current stimulators are effective in delivering nGVS to vestibular afferents, demonstrating the potential of nGVS as a prosthetic technique [25]. Modulating anodal and cathodal currents provides a means of modulating the firing rate of vestibular afferents [24]. This technique has been shown to improve balance control, roll-tilt perception and postural response in healthy subjects [26,27,28]. Moreover, it has been demonstrated that nGVS elicits significant improvements in standing balance, gait parameters and vestibulospinal function following nGVS trreatment [28]. The ameliorating effects are a result of stochastic resonance (SR), a mechanism in which weak input signals in a nonlinear system can be strengthened by a small amount of ‘noise’ [28]. This can boost subthreshold stimuli, effectively lowering the detection threshold of the system [29]. 

The function of the five vestibular end organs and vestibular hair cells can be modulated by nGVS, further indicating its clinical potential as an effective technique to improve balance [24]. It has also been demonstrated that using different configurations of stimuli can assist balance in both the sagittal and frontal planes [30]. However, the mechanism of nGVS means that patients with no residual vestibular function, although few in number, would fail to benefit from this treatment [31]. 

### 1.3. Research Aims

The primary purpose of this review was to systematically evaluate the effectiveness of vestibular prostheses on behavioural outcomes related to balance in humans. The secondary aim was to evaluate, compare and appraise the relative potential of both classes of vestibular prostheses. The results address a research gap by investigating and comparing the extent to which both techniques affect behaviours related to balance in humans.

## 2. Methodology 

**Figure 1:**
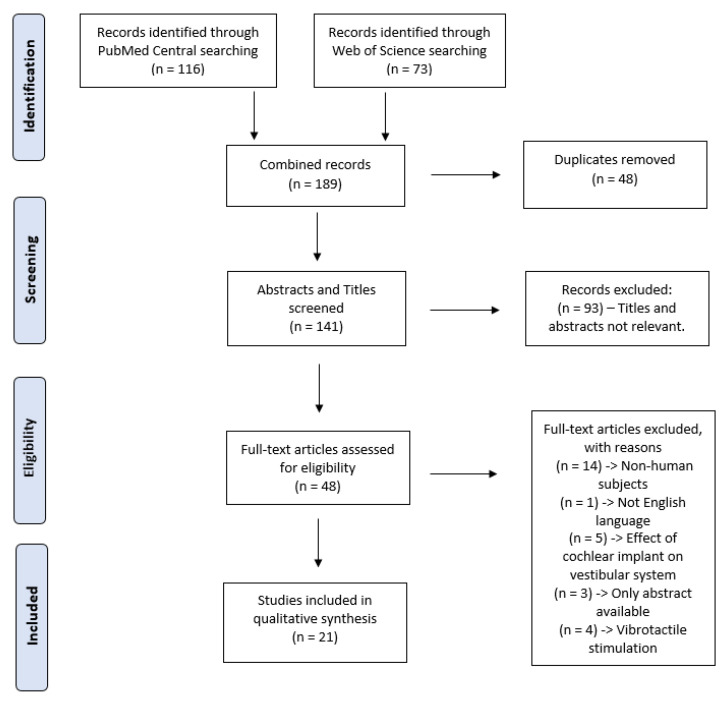
The PRISMA flowchart representing the study selection process.

This systematic review was conducted in adherence to the Preferred Reporting Items for Systematic Reviews and Meta-Analyses (PRISMA) guidelines [32] (Figure 1) which has been used in previous systematic reviews [33,34].

### 2.1. Search Strategy

A literature search was conducted on 10/2/19 using two databases: Web of Science and PubMed Central. Search terms were combined using the Boolean Operators ‘AND’, ‘OR’ and ‘NOT’ to construct a search algorithm. The following search was applied to both databases:

#1 vestib* OR ménières OR ‘bilateral vestibulopathy’ OR BV OR BVD OR BVL

#2 prosthe* OR implant* OR gyroscop* OR accelerometer* OR ‘noisy galvanic vestibular stimulation’ OR nGVS OR galvan* OR GVS

#3 ‘vestibular-ocular reflex’ OR VOR* OR gait OR balance OR postur* OR walking OR dizziness OR stabilis* OR tilt OR Outcome OR performance

#4 #1 AND #2 AND #3

Following this, the species was restricted to ‘Human only’ in the PubMed database, and the ‘Full-Text available’ filter was applied in both databases. 

### 2.2. Selection Process

Following the search, the primary reviewer screened and removed duplicate and follow-up papers. A two-stage selection process was then undertaken; sequential screening of the title/abstract followed by the full-text articles was conducted. Only articles that were deemed potentially relevant following the title/abstract screening were selected for full-text evaluation. Finally, the papers included were selected following screening against the subsequent exclusion criteria: (i) Not conducted on humans, (ii) did not include a device directly affecting the vestibular system; (iii) no quantified biomechanical behaviour related to balance reported, (iv) not in the English language, (v) no access to a full-text article, and (vi) not published in a peer-reviewed journal. The full-text screening was conducted by the reviewers and the main reason for each exclusion was listed. Articles published by journals that did not have a scientific journal rating (SJR) of either Q1 or Q2 were excluded. This was a means of ensuring that the studies reviewed were of an appropriate standard in their relative scientific domain [35].

### 2.3. Data Extraction

Data regarding the number of participants, year of publication, sex, age, outcome(s) measured, side effects reported, statistical significance of results, pre-interventional vestibular function, intervention details and implantation process were all deemed significant and extracted when possible. Where appropriate, these data were summarised and tabulated. Published data quantifying the effects of artificial vestibular stimulation have only recently become available. A high proportion of the studies published are first-of-their-kind, feasibility or pilot trials. These studies often have the aim of proving a concept, testing a prototype or determining the safety of a new intervention. Inevitably, these exploratory trials display extensive clinical and methodological heterogeneity. As a consequence of this heterogeneity, pooled data analysis or meta-analysis were considered inappropriate for this review. Instead, the results and methodology were appraised narratively. 

## 3. Results

### 3.1. Search Results

The search of the PubMed database yielded 116 studies, following application of the prementioned filters. The same search yielded 73 studies from the Web of Science database. The removal of duplicate articles resulted in a total of 113 non-duplicate papers. After the screening of the abstracts, this was reduced to 49 papers which were eligible for full-text review. Twenty-one studies were selected following a full-text review, as the other 28 studies conflicted with one or more of the predetermined exclusion criteria. Finally, the SJR of each paper was retrieved. All 20 papers were found to be from Q1 or Q2 journals, affirming full justification of their inclusion. All abstracts were summarised during screening and are displayed. 

### 3.2. Study Characteristics

The studies included were conducted on various populations. These included: adults with BVD (*n* = 8), healthy adults (*n* = 6a), adults with BVD and bilateral deafness (*n* = 3), adults with unilateral Ménière’s disease (*n* = 2), adults with bilateral Ménière’s disease (*n* = 1), and healthy pilots (*n* = 1) as displayed in Table 1. A total of 19/21 studies were published in journals with the rating of Q1 on 15/03/2019. Two journals were found to have a ranking of Q2 [15,21]. In general, the sample size was small; three studies only had one participant [36,37]. Moreover, only seven out of twenty-one studies had more than 10 subjects (Table 2). All papers reported participant age and sex, excluding the study by Peterka et al. [26] which failed to specify either. All reports with more than one participant included people of both sexes. Only one study had a randomized controlled trial experimental design [26]. The most common study design was a single-arm crossover trial, with randomization to determine the order in which the patient received either a placebo stimulation or the defined intervention. 

### 3.3. Galvanic Vestibular Stimulation (GVS) 

A total of 11 studies investigated the use of GVS to modulate vestibular function [16,25,26,27,28,31,38,39,40,41,42]. Only five of these were conducted on participants with BVD, with the rest conducted on healthy patients. In general, the studies conducted on healthy participants had a larger sample size, ranging from 9–58 individuals. Conversely, experiments on patients with BVD were generally smaller and ranged from 1–13.

Excluding a 2001 study by Scinicariallo et al. [28], all studies were conducted after the year 2012, with more than half published between 2016–2018 [Table 2]. No commercial or financial conflicts of interest were disclosed in any of these studies.

Application technique was very similar in all studies. All stimulation was delivered bilaterally to electrodes placed on the mastoid processes behind each ear. Electrode size was roughly 2 cm × 2 cm in all studies apart from Keywan et al. in which the electrodes were 4 cm × 6.4 cm [27]. Only five trials explicitly stated the use of a portable, battery-powered stimulator [16,25,26,40,42]. The size and weight of the portable stimulator was only mentioned in two studies which both used the same model (112 mm × 67 mm × 28 mm, 200 g) which was worn attached to the waist [25,42]. Two studies applied constant GVS [26,28]. The rest applied GVS as zero-mean white noise (nGVS). The optimal stimulus intensity was not established for each individual patient in four studies [26,28,38,39]. Two of these studies selected the intensity used based on generalised findings of previous reports [38,39]. The other two papers failed to justify their choice of stimulus intensity [26,28]. Iwasaki et al. were the first group to use graded intensities of nGVS stimulation to elucidate the optimal stimulus intensity for improving postural stability while standing [40]. This protocol was followed in four proceeding studies. These, unlike the study by Iwasaki et al. which had the primary intention of finding the optimal amplitude, went on to use this optimal stimulus intensity as a tool to explore different biomechanical effects of nGVS on balance [16,25,27,42]. The mean amplitude optimal stimulus intensity found for patients with BVD ranged from 454 (±55) to 456 μA (±82). In healthy adults, lower optimal amplitudes were observed, and these ranged from 134.6 (±86) to 281 μA (±40). 

Postural response in the form of body sway was the most common behaviour measured, reported in 9/11 of the papers (Table 3). This required a two-legged stance task in which the centre of pressure (COP) was measured and any changes in the following three parameters were recorded: COP velocity, COP envelopment area and the root mean square (RMS) of the COP. 

The task was performed with eyes closed in 2/8 cases to minimise visual contribution to balance [25,42]. Peterka et al. [26] constrained subjects to sway as a ‘single-link inverted pendulum’, only allowing sway rotation around a single-axis located at ankle level. This simplified model was aimed to reduce proprioceptive input to balance control [26]. Seven of these studies quantified the change in sway specifically in the mediolateral direction, with only one study measuring sway in both the mediolateral and anteroposterior axes [38]. All studies measuring postural sway (in both healthy subjects and those with BVD) discovered statistically significant improvement in at least one of the parameters measured when nGVS was applied. Only four studies used a sham (0mA) stimulation as a placebo [16,38,40]. Significant improvement in ≥1 of the parameters measured was consistent across all of these studies, despite heterogeneity in both methodology and population characteristics (Table 3). Taken together, these trials present strong evidence that nGVS has at least some positive effect on static balance control, despite examples of poor study quality. 

Only one paper examined the effect of nGVS on dynamic balance as opposed to static balance [16]. This study looked at changes in dynamic walking stability in 13 patients with BVD, comparing the effect of nGVS to a sham stimulation placebo. Eight standard gait parameters were measured. Stride time (*p* < 0.041), phase co-ordination (*p* < 0.013) and base of support (*p* < 0.037) were all significantly improved by nGVS, which was applied at the optimal intensity of individual patients. Decreased stride-to-stride fluctuations in the ML plane and improved subjective ratings of balance while walking was also reported. The effects were most pronounced at slow walking speeds. This was a Class IV evidence study. Patient blinding to stimulation protocol and the use of a placebo shows that nGVS improves gait performance in patients with BVD [16].

Vestibular motion perception, another key mediator of balance control, was tested in one paper using a motion platform and sensor [27]. After determination of individual optimal nGVS intensities, 15 healthy subjects performed direction-recognition tasks, with alterative application of nGVS and sham stimulation. At 0.5 and 1.0Hz stimulation, direction-perception thresholds were significantly reduced by nGVS (*p* < 0.05). The use of a sham placebo, randomized order of interventions and participant blinding to protocol, all support the hypothesis that roll-tilt vestibular motion perception is enhanced by nGVS.

Only two studies directly compared behaviours between healthy participants and participants with BVD [26,40]. Peterka et al. demonstrated that a patient with BVD was able to maintain balance on a surface that was sway referenced in the mediolateral plane only with the application of GVS [26]. Subsequently, it was demonstrated that GVS application at a different frequency could remove the ability of a healthy subject to use their vestibular system for balance control. The sample size of one patient in each group is not clearly justified and questions the validity of the evidence. A team led by Iwasaki recently observed that nGVS improved body balance in both patients with BVD and healthy subjects [40]. All three parameters measured increased in 76% of healthy subjects and 91% of patients with BVD. This larger sample size and predetermination of the optimum stimulus amplitude for each participant before testing increased the reliability of the results. However, one limitation was the difference in stance task methodology between the two groups. Initially, a foam rubber platform was used to increase sensitivity, however, patients with BVD were unable to balance on this with their eyes closed so they performed the tasks without the platform [40]. 

Two studies investigated the sustained effect of vestibular enhancement following nGVS [25,42]. Fujimoto discovered that the ameliorating effect of nGVS on postural stability had not significantly decreased 2 h after stimulus cessation [25]. However, this study did not have a placebo. It is, therefore, possible that habituation to the posturography methods could have affected the results. Uemera et al. reported an improvement in COP velocity in the anteroposterior axis for up to 6 h after stimulus cessation (*p* < 0.05) [42]. This study also measured subjective improvement in the balance of the BVD patients, which was also significantly higher up to 6 h after stimulation (*p* < 0.05) [42]. This study was a single-arm trial and it is possible that the subjective improvement scores were attributable to the placebo effect. However, significant improvement in postural control due to stimulation extended well beyond 2 h. A total of seven papers allowed a time period of under 10 min to ‘recover from the effect of nGVS’ [16,27,31,38,39,40,41]. The findings of Uemura and Fujimoto call into question the methodological validity of these trials, as they proved it can take far longer for the effects to subside.

### 3.4. Vestibular Implant 

A total of 10 papers reported on the effect of direct vestibular stimulation using implanted electrodes [14,15,21,36,37,43,44,45,46,47]. Only 20% of studies had a sample size >5 patients [43,44]. All papers were published between 2011 and 2017 [Table 2]. Financial contributions from companies that produce cochlear implants (Cochlear Ltd./Med-El) were reported in three studies and could be considered a potential source of funding bias [15,36,47]. No other conflicts of interest were described.

A variety of surgical techniques were used for electrode implantation and positioning [Table 3]. Five studies used an intralabyrinthine ampullar approach to position electrodes inside the SCCs, adjacent to the ampullae [15,36,37,46,47]. Conversely, an extralabyrinthine approach was used by Guyot et al. to position a single electrode in the vicinity of the posterior ampullary nerve [21]. Three studies were conducted on a patient pool containing implants that had been inserted using both extralabyrinthine or intralabyrinthine techniques [43,44,45]. However, no subgroup analysis was conducted to investigate a potential difference in the stimulation effect of the two methods in any study [14]. In these reports, both hearing and residual vestibular function were lost after implantation [36,46]. No other adverse effects were reported apart from brief nystagmus following electrode activation in one study [21]. In summary, both surgical techniques have been shown to successfully implant electrodes that facilitate artificial stimulation, but post-implantation hearing preservation was not been reported in humans in any included study. The biomechanical effect of vestibular stimulation on eye movement was the most commonly reported behaviour measured [Table 3]. This was examined in 80% of studies, however, there was variation in both the specific outcome reported and the study methodologies [15,21,36,37,43,45,46,47]. Three of the eight were feasibility trials, each conducted on just one participant [21,36,37]. In 2011, Guyot et al. showed that humans can adapt to steady state vestibular stimulation [21]. Moreover, once adaptation was complete, modulation of amplitude or frequency of stimulation elicited smooth predictable eye movements [21]. The electrode activated was placed in the vicinity of the posterior ampullary nerve. Stimulating the PAN would normally cause vertical eye movements, in contrast, horizontal eye movements were observed [21]. This was suggested to have been caused by current spread to other vestibular end organs [47]. Following this, van de Berg et al. trialled a modified surgical approach targeting stimulation of the ampullae via an intralabyrinthine approach [37]. The subject was a female patient who had experienced bilateral deafness and vestibular dysfunction for over 20 years. Ampullar stimulation was successfully conducted, eliciting VORs in both the vertical and horizontal components [37]. This experiment was conducted preoperatively and all testing was done while the patient was under general anaesthesia [37]. The study acknowledged the effect that the anaesthesia had on results, although could not quantify it. Finally, a 2014 study by Golub et al. showed the feasibility of prosthetic implantation of the SCCs, finding that implantation was well tolerated in a human subject with uncontrolled Ménière’s disease [36]. Canal-specific controlled eye movements were also elicited [36]. To summarise, these feasibility trials established three important developments: direct stimulation of the ampullae is surgically feasible, amplitude modification following adaptation can induce bi-directional eye movements and a vestibular implant can be well tolerated [18]. 

Guinand et al. similarly found that direct electrical stimulation is a safe and effective method of vestibular stimulation [43]. This trial provided much stronger evidence with a sample size of 11 patients and with some implants having been monitored over a period of eight years. No adverse effects were observed and the mean eye movement velocity after stimulation was found to be within the range required for everyday dynamic activities in one patient (26°/s) [43]. One study measured the effect of a broad spectrum of frequencies on VOR response in order to emulate the higher frequency range caused by dynamic movements [45]. A video head impulse test was used to measure improvements in VOR across a large frequency range and this was the only method that measured eye movement in three dimensions. Although it was shown that it is possible to restore VOR across this frequency range, high levels of asymmetry in response were observed. Excitatory stimulation caused a significantly higher response than inhibitory stimulation [45].

Phillips et al. measured postural responses during stimulation of the vestibular end organs [15]. A total of four patients with long-term Ménière’s disease were implanted with a modified cochlear implant (Cochlear Ltd.) which placed an electrode in close proximity to each of the three ampullae using an intralabyrinthine approach. A two-legged stance task was performed with eyes open and eyes closed. Patients were blinded to the procedure of the approach. Two seconds of stimulation was shown to induce whole-body sway in both the ML and AP planes. Furthermore, stimulation current amplitude was shown to correlate with peak sway amplitude (*p* < 0.05) [15]. This study measured the results against control of results from the same trial without stimulation, although it was unclear whether patients were blinded to the protocol. This trial was the first in humans to show postural responses to artificial stimulation that were specific to the SCC stimulated [15].

The only study of the effect of a vestibular implant on dynamic stability in humans was published by Guinand et al. in 2016 [44]. It measured visual acuity (VA) using Sloan letters exhibited on a computer screen as patients walked on a treadmill at different speeds. Six patients, with BVD of various aetiologies, were implanted with a modified cochlear implant and electrodes were implanted with either an extralabyrinthine or intralabyrinthine approach depending on the patient. When the system was on, visual acuity whilst walking was significantly increased (*p* < 0.001) compared to results obtained using sham stimulation. VORs were not recorded in this experiment. If VORs had been quantified, it could be determined whether the improvements in VA were as a result of VOR restoration. This was the first successful demonstration of improvement in a dynamic task that could be considered close to reality [44].

## 4. Discussion and Conclusions

All 21 studies showed that artificial vestibular stimulation resulted in a significant improvement in at least one behaviour related to balance control. Most commonly, this was VOR restoration (for vestibular implants) and postural sway (for nGVS) (Table 2 and Table 3). Improvements in dynamic visual acuity, postural sway control, vestibulospinal function and gait speed were also shown to improve after vestibular stimulation [14,31,40,44]. Moreover, a randomised control trial showed improved balance control was also reported in healthy patients after nGVS [39]. In the context of the primary research question, the literature reviewed showed that some biomechanical balance-related behaviours were significantly improved by artificial vestibular stimulation. Notably, VOR response and dynamic visual acuity while walking were both restored to near-normal levels [44,45]. In general, populations studied were both small and heterogeneous. Moreover, the inter-study variation in the behaviour measured was substantial (Table 2 and Table 3). However, the unanimity amongst studies regarding a statistically significant improvement in the respective behaviour measured means that there is moderate evidence supporting the effectiveness of both techniques in balance restoration. This effectiveness could neither be quantified nor compared due to heterogeneity in methodology. Developmental progress of both techniques would benefit from consensus on the most effective delivery technique. Stimulation using nGVS shared a relatively consistent methodology across all studies. Nevertheless, the results of the review showed that failure to establish optimal stimulus amplitude before testing correlated with reduced stimulation effect compared with trials that individually ascertained this [39]. A common limitation was the testing of eye movements in two-dimensions; the video head impulse test was shown to be a more sensitive technique and should, therefore, be considered for use in future studies [45].

Conversely, there was significant heterogeneity in both the surgical implantation process, behaviour measured and stimulation technique of trials on vestibular implants [Table 3]. This meant that it was difficult to draw conclusions in cases where results differed, or across the two classes of the prosthesis. Accordingly, part of the secondary aim of the project, which was to compare the relative efficacy of both classes of the prosthesis, could not be adequately addressed. In the future when larger populations are available, subgroup analyses should be conducted to assess the effect of factors such as surgical approach and stimulation intensity on the outcome.

The only two studies that implanted on patients with hearing intact both caused severe, irreversible hearing loss [36,40]. The majority of patients with BVD and other balance-affecting diseases are not also deaf [23]. Further research should be conducted exploring the potential for improved surgical techniques that retain auditory function. As a practical prosthetic device, the two techniques have different strengths. GVS is shown to have a strong and long lasting post-stimulatory effect meaning constant activation may not be necessary [42].

Little evidence of bias was found in the studies included. However, funding from companies that produce cochlear implants (Cochlear Ltd. & MedEl) could be considered a potential funding bias as most vestibular implants are modified from existing cochlear implants. Data analysis was adequately conducted and reported across all studies. This review confirms the potential of vestibular implants for improving balance in humans, but more studies into the biomechanical effects elicited are needed to optimise the design before large-scale trials can be conducted.

In conclusion, this review found that sufficient evidence to indicate that both vestibular implantation and the delivery of noisy galvanic stimulation can improve behavioural outcomes related to both static and dynamic balance. Consequently, they have the potential to be used as a vestibular prosthesis to improve balance in humans. However, for patients with no residual function, nGVS will have little-to-no effect due to its mechanism of action. Differences in outcome measured, participant population and study methodology made it difficult to draw overall conclusions regarding the relative potential of both mechanisms to each other. Based on the findings of this review, suggestions for future research include: (i) standardisation of nGVS application methodology, including the ascertaining of individual optimal stimulus intensities before testing; (ii) subgroup analysis of larger data pools, when they become available, to determine the relative effectiveness of different implant designs; (iii) application of nGVS was shown to have no adverse effects, even in the long term. This warrants research on the long-term effect of a prosthetic device on fall-reduction and patient quality of life; (iv) more research needs to be conducted on the potential for hearing preservation following implantation; and (v) the use of nGVS as a treatment for other diseases affecting balance, such as Parkinson’s disease, should be explored.

## Figures and Tables

**Table 1:** A summary of the measured behaviours, methods and results of the reviewed published studies.

Reference	Behaviour(s) Measured	Methods	Summary of Results
[16]	8 gait parameters	Walking balance tested at 3 speeds, eyes closed	nGVS improved: -Stride time, stride length, base of support, phase coordination index (*p* < 0.05)-Benefits most pronounced during slow walking.
[25]	Postural stability	Two-legged stance task, eyes closed	nGVS improved: -Postural stability (*p* < 0.01) (measurements were taken 2 h after stimulation)
[38]	Postural stability	Two-legged stance task, eyes open	nGVS improved: -ML mean velocity(*p* < 0.01)-AP mean velocity (*p* < 0.01)-Sway path length (*p* < 0.01)
[39]	Postural Sway	Two-legged stance task, eyes open	nGVS improved: -Postural sway (*p* < 0.01)
[40]	Postural stability	Two-legged stance task, eyes open	nGVS improved postural stability in:-76% of healthy subjects (*p* < 0.01)-91% of patients with BVD (*p* < 0.01)
[27]	Vestibular motion perception during roll rotations	Direction-recognition tasks on a motion platform, eyes closed	nGVS Improved:-Direction-recognition thresholds at 0.5 μA and 1.0 μA (*p* < 0.05)-No significance at 0.2 μA (*p* > 0.05)
[26]	ML balance control	Balance tested on the sway-referenced surface, eyes closed	nGVS improved: ML balance control (*p* < 0.05)
[41]	ML body sway	Two-legged stance task, eyes open	nGVS caused:-ML body sway in both groups(*p* < 0.01)-The response was lower in pilots compared to the general population (*p* < 0.001)
[31]	Vestibulospinal function	Body motion response to stimulation, eyes open	nGVS improved: -vestibulospinal function in 90% of patients (*p* < 0.05)-No response to nGVS in patients with complete BVD
[28]	ML body sway	Two-legged stance task on motorised platform, eyes open	GVS improved:-ML sway latency (*p* < 0.01)-ML sway amplitude (*p* < 0.01)
[42]	Postural stability	Two-legged stance, eyes closed	nGVS improved: -AP latency and sway, even 6-h post-stimulation (*p* < 0.01)

**Table 2:** A summary of the population characteristics and scientific journal impact of papers published investigating the effect of artificial vestibular stimulation on behaviours related to balance control in humans.

Reference	Journal (SJR Quartile)	Participant Details Provided	Description of Vestibulopathy
[16]	Neurology (Q1)	13 Adults (5 female), mean age = 50.1 ± 5.5 years	BVD
[25]	Scientific Reports (Q1)	30 Adults (13 Female), mean age = 67.0 ± 0.03 years	Healthy
[36]	Otology and Neurotology (Q1)	1 Adult (Male), age = 56 years	Ménière’s disease
[43]	ORL (Q1)	11 Adults (3 Female), mean age = 60.2 years	BVD and unilateral or bilateral hearing loss
[44]	Frontiers in Neuroscience (Q1)	6 Adults (2 Female), mean age = 58 years	BVD and unilateral or bilateral hearing loss
[45]	Journal of Frontiers in Neurology (Q1)	3 Adults (1 Female), mean age = 53.3 years	BVD
[21]	Annals of Otology, Rhinology and Laryngology (Q2)	1 Adult (Male), mean age = 69 years	BVD and bilateral hearing loss
[38]	Journal of Neuroengineering and Rehabilitation (Q1)	32 Adults (25 Female), mean age = 75.8 ± 0.8 years	Healthy
[39]	Brain Stimulation (Q1)	18 Adults (12 Female), mean age = 21.8 ± 1.1 years	Healthy
		24 Adults (11 Female), mean age = 22.2 ± 1.8 years	Healthy
		16 Adults (10 Female), mean age = 21.9 ± 1.2 years	Healthy
[40]	Neurology (Q1)	21 Adults (10 Female), mean age = 38.7 ± 2.6 years	Healthy
		11 Adults (5 Female), mean age = 46.4 ± 5.2 years	BVD
[27]	Frontiers in Neurology (Q1)	15 Adults (7 Female), mean age = 25.1 ± 1.7 years	Healthy
[14]	Frontiers in Neurology (Q1)	3 Adults (2 Female), mean age = 63.7 years	BVD, unilateral or bilateral hearing loss
[46]	Neurophysiology (Q1)	4 Adults (2 Female), mean age = 67 ± 9 years	Unilateral Ménière’s disease
[26]	Proceedings of the Annual International Conference of the IEEE Engineering in Medicine and Biology Society, EMBS (Q1)	2 Adults (Sex and age unspecified)	1 Healthy, 1 BVD
[15]	Experimental Brain Research (Q2)	4 Adults (2 female), mean ag e = 67 ± 9 years	Unilateral Ménière’s disease
[41]	BioMed Research International (Q1)	12 Students, mean age =23.8 years	Healthy
		12 pilots, mean age =25.3 years	Healthy
[31]	Journal of neurology (Q1)	12 Adults (6 female), age unspecified	2 complete BVD 10 partial BVD
[28]	Biological Cybernetics (Q1)	9 Adults (6 female), mean age =22 years	Healthy
[42]	Frontiers in neurology (Q1)	13 Adults (5 female), mean age =63.1 ± 4.0 years	BVD
[37]	Frontiers in neurology (Q1)	1 Adult (female), age =21 years	Complete BVD and bilateral hearing loss
[47]	Frontiers in neurology (Q1)	4 Adults (2 female), mean age =58.8 years	BVD

**Table 3:** The characteristics of studies investigating artificial electrical stimulation of the human vestibular system via implanted electrodes. A concise summary of prosthetic design, behaviour(s) measured, key results reported and adverse effects observed.

Reference	Details of Prosthesis	Behaviour(s) Measured	Summary of Results	Adverse Effects Reported
[14]	Modified cochlear implant, intralabyrinthine ampullar approach	VOR response	Activation of LAN electrode elicited VOR response (*p* < 0.01)	N/A
[36]	Modified cochlear implant, intralabyrinthine ampullar approach	Electrically evoked eye movements	Stimulation of canal-specific eye movements successful in 2/3 electrodes	Severe loss of horizontal canal and auditory function
[45]	Modified cochlear implant, both surgical approaches used	High-frequency aVOR response	aVOR restored in a broad frequency range	N/A
[44]	Modified cochlear implant, both surgical approaches used	Visual acuity in dynamic conditions	VA improved when the implant was turned on (*p* < 0.001)	N/A
[43]	Modified cochlear implant, both surgical approaches used	VOR response	Stimulation of canal-specific eye movements successful in 17/24 available electrodes.	N/A
[21]	Modified cochlear implant, extralabyrinthine approach	Electrically evoked eye movements	Stimulation of PAN caused smooth canal-specific eye movement	Initial strong nystagmus abated after vestibular adaptation
[15]	Modified cochlear implant, intralabyrinthine ampullar approach	Postural responses	2 s of electrical stimulation of each SCC all elicited sway response	Reduced vestibular function in implanted ear
[46]	Modified cochlear implant, intralabyrinthine ampullar approach	VOR response	Stimulation of ampullary nerves caused a proportional VOR response	Hearing and vestibular loss in implanted ear
[37]	Modified cochlear implant, intralabyrinthine ampullar approach	Electrically evoked eye movements	Ampullar stimulation evoked eye movements in a patient after 20 years of no vestibular function	N/A
[47]	Modified cochlear implant, intralabyrinthine ampullar approach	VOR	Artificial stimulation of the vestibular nerve branches interacts with residual vestibular function	N/A

## Abbreviations

SCC	Semi-circular canal
BVD	Bilateral vestibular dysfunction
VOR	Vestibular-ocular reflex
aVOR	Angular vestibular-ocular reflex
RMS	Root mean square
nGVS	Noisy galvanic vestibular stimulation
LAN/PAN/SAN	Lateral/Posterior/Superior ampullary nerve
COP	Centre of pressure
VA	Visual acuity
QALY	Quality-adjusted life year
ML	Mediolateral
AP	Anteroposterior
ANOVA	Analysis of variance
Q1	Quartile one
Q2	Quartile two
